# Cytomics of the tumour microenvironment: therapeutic targeting (keynote lecture)

**DOI:** 10.1186/1476-9255-12-S1-O10

**Published:** 2015-04-16

**Authors:** Paul Smith

**Affiliations:** 1 Institute of Cancer and Genetics, Cardiff University, CF14 4XN, UK; 2 Oncotherics Ltd, Shepshed, Leicestershire LE12 9NP, UK

## 

There is a growing appreciation of the influence of both the tumour microenvironment and the dynamic nature of the cellular micro-community in determining tumour progression and therapeutic resistance. Changes in cell heterogeneity, resulting from time-varying changes in the microenvironment, present complex challenges for cancer therapy. For example, the tumour micro-community comprises multiple levels of cellular interactions overlaid with pervading influences such as variation in the levels of oxygenation. Molecular targeting combined with agents that target the microenvironment offer novel approaches. The presentation focused on two examples supported by cytometric techniques. First, dissecting the influences of cellular interactions on tumour progression-related cell behavior. An *in vitro*, non-adherent small cell lung cancer (SCLC) cell model system enabled the engineering of cellular micro-communities. SCLC cells show surface expression of a potential drug target, polysialylated neural cell adhesion molecule (NCAM; NCAM1 or CD56). The extensive addition of polysialic acid (polySia) to NCAM in SCLC decreases membrane apposition, cell adhesion and enables cell migration. Cytometric studies reveal heterogeneity in the expression of polySia in SCLC cell populations that can be related to proliferation potential both *in vitro* and *in vivo*. In constructed 3-D micro-communities, polySia expression was driven in non-expressing variants by the presence of over-expressing subpopulations. This suggests that a degree of homeostasis operates with implications for polySia-NCAM-targeted therapies. The second example addressed the targeting of hypoxia for therapeutic advantage using a novel hypoxia activated prodrug (HAP) technology. Hypoxia in tumour regions, arising from loss of vascularity, generates drug resistant phenotypes. Targeting hypoxic tumour cells using HAPs has been previously attempted using a diverse group of chemicals based mainly on structures with alkylating functionality. Crucially most of these agents are reduced in single electron steps by a number of enzymes including cytochrome P450 reductase. This produces prodrug conversion to species that bind covalently to DNA. Critically, the cytotoxins generated do not persist such that targeted cells upon re-oxygenation may contribute to tumour regrowth. We have developed the concept of a unidirectional hypoxia activated prodrug (uHAP), building upon experience with di-N-oxides of tertiary amine anthraquinones, to develop the novel agent OCT1002. The uHAP represents a distinct class of HAP, reduced to a cytotoxic species through an irreversible two-step process, each involving a two-electron reduction. The bioactive reduction products inhibit DNA processing and topoisomerase II activity, selectively causing cancer cell death. The presentation explored the application of cytometric methods to reveal the mechanisms of action of OCT1002 and to provide an insight into the targeting of hypoxic regions of prostate and pancreatic cancers. The above examples highlight the importance of cell-based approaches in the development of therapeutic strategies that address changing patterns of target presentation in different cellular microenvironments. [PJS acknowledges the contributions of Prof. Rachel Errington (Cardiff University); Prof. Stephanie McKeown (University of Ulster) and Robert Falconer (Bradford University)].

